# Effect of tranexamic acid in spine surgeries: a systematic review and network meta-analysis

**DOI:** 10.3389/fsurg.2025.1550854

**Published:** 2025-04-11

**Authors:** Sung Ryul Shim, Sangah Han, Ji Hun Jeong, Inhwan Hwang, Yonghan Cha, Chunhwa Ihm

**Affiliations:** ^1^ Department of Biomedical Informatics, College of Medicine, Konyang University, Daejeon, Republic of Korea; ^2^ Konyang Medical Data Research Group-KYMERA, Konyang University Hospital, Daejeon, Republic of Korea; ^3^ Department of Blood Management Services, Daejeon Eulji Medical Center, Eulji University, Daejeon, Republic of Korea; ^4^ Department of Laboratory Medicine, Daejeon Eulji Medical Center, Eulji University, Daejeon, Republic of Korea; ^5^ Department of Hematooncology, Daejeon Eulji Medical Center, Eulji University, Daejeon, Republic of Korea; ^6^ Department of Orthopaedic Surgery, Daejeon Eulji Medical Center, Eulji University, Daejeon, Republic of Korea

**Keywords:** systematic review, network meta-analysis, tranexamic acid, blood transfusion, spine surgeries

## Abstract

**Background:**

Severe blood loss during spine surgery increases the need for blood transfusion. Transfusion carries the risks of infection, complications, and postoperative morbidity; therefore, minimizing these risks is crucial for all surgical patients.

**Methods:**

A comprehensive literature search was conducted in PubMed, Cochrane, and EMBASE to find studies examining the effect of tranexamic acid (TXA) on spine surgeries in patients who received blood transfusion. We used the mean difference (MD) and 95% credible intervals (CrI) to analyze continuous outcomes, such as intraoperative blood loss, postoperative blood loss, hemoglobin drop, and length of hospital stay. To evaluate categorical outcomes, such as blood transfusion rate and complication rate, the odds ratios (OR) and 95% CrI were determined.

**Results:**

A total of 38 randomized controlled trials were included, evaluating six outcomes across 10 treatment groups. Low-dose intravenous (IV) TXA combined with temperature intervention (15 mg/kg) significantly reduced intraoperative blood loss compared with placebo [MD: −112.0; 95% CrI: −211.0 to −14.9, surface under the cumulative ranking curve (SUCRA): 78.37%]. The administration of more than two doses of TXA significantly reduced intraoperative blood loss (MD: −101.0, 95% CrI: −161.0 to −44.1, SUCRA: 77.65%) and postoperative blood loss (MD: −177.0, 95% CrI: −275.0 to −92.4, SUCRA: 85.66%) compared with placebo. Both treatments significantly impacted the hemoglobin drop and blood transfusion rate.

**Conclusions:**

Low-dose IV TXA with temperature intervention and the combined use of TXA significantly improved blood loss, hemoglobin drop, and blood transfusion rate during spine surgeries. Further studies involving larger populations are warranted and should be carefully designed to determine the potential risk of complications.

**Systematic Review Registration:**

www.crd.york.ac.uk/prospero/display_record.php?ID=CRD42024531557, identifier: CRD42024531557.

## Introduction

Blood transfusions are often necessary in many surgical procedures, with cardiovascular and orthopedic surgical procedures posing the highest risks of blood transfusion (1). The number of spine fusion procedures performed in the United States increased from 54, 000 to 350, 000 between 1993 and 2007 (2). In Australia, simple lumbar fusion rates increased from 1.3 to 2.8 per 100,000 people, while complex fusion rates increased from 0.6 to 2.4 per 100,000 people from 2003 to 2013 (3). These trends are likely related to the aging population and increased average life expectancy. Elderly individuals often develop various medical comorbidities, including reduced bone mass density, osteoporosis, decreased mobility, spine degeneration and deformities, poor balance, and a higher risk of falls (4). Evidence shows that the blood transfusion rate during adult spine fusion surgery ranges from 50% to 81%. Most studies on spine surgery reported that the amount of blood loss requiring blood transfusion ranges from 650 to 2,839 ml per case (5).

Orthopedic surgeries, including spine surgery, often result in significant blood loss. Spine surgery, which involves bone resection and extensive dissection of soft tissues, is particularly associated with substantial perioperative blood loss. This can be attributed to large wound areas, prolonged operative times, and the involvement of abundant cancellous bone. Although the amount of perioperative blood loss may significantly vary among procedures depending on surgical and nonsurgical factors, it remains a major concern during spine surgery (6).

Blood transfusions increase the risk of infections, complications, coagulopathy, and postoperative morbidity (7, 8). These risks also contribute to prolonged hospital stays and negatively impact quality of life. Therefore, minimizing these risks is essential for all patients undergoing surgery. Various strategies have been employed in spine surgeries, including the use of recombinant erythropoietin and intravenous iron preparations for the preoperative correction of anemia. Additionally, hemostatic agents, autologous blood transfusions, cell savers, and tranexamic acid (TXA) have been used to reduce intraoperative and postoperative blood loss.

TXA is a synthetic derivative of the amino acid lysine that exerts its antifibrinolytic effect by blocking the lysine-binding sites on plasminogen molecules, thereby inhibiting the interaction of plasminogen and plasmin's heavy chain with lysine residues on fibrin surface (9). Reducing blood loss and blood transfusion rate is the ultimate goal of surgery. Various doses and methods of TXA administration have been explored. For instance, TXA can be administered intravenously, orally, topically, or in combination. Several studies have been conducted to determine the optimal approach (10, 11). Recently, temperature control methods for maintaining normal body temperature have also been investigated (12). However, despite these efforts, determining the most appropriate approach for administering TXA remains controversial. Therefore, no consensus or official guidelines have been established.

Although previous meta-analyses have been conducted (13), recent randomized controlled trials (RCTs) have emerged since then. Among them, providing evidence-based medicine for comprehensive decision-making, particularly incorporating new methods such as temperature intervention, remains essential. Many surgeons perform fusion surgery as their personal preference (14). Concerns also persist regarding the use of TXA due to the potential risk of myocardial infarction (MI), stroke, deep vein thrombosis (DVT), and pulmonary embolism (PE) (6, 15).

This systematic review and network meta-analysis (NMA) evaluated RCTs to determine the optimal method of administering TXA and its effects on blood loss, blood transfusion rate, complication rate, and length of hospital stay.

## Materials and methods

This study was registered in the PROSPERO database (registration number: CRD42024531557) and conducted in the Preferred Reporting Items for Systematic Reviews and Meta-Analyses (PRISMA) statement (16).

### Data sources and literature search

Three independent authors (SR Shim, S Han, and C Ihm) conducted a comprehensive literature search in PubMed (https://pubmed.ncbi.nlm.nih.gov/), Cochrane (https://www.cochranelibrary.com/search), and EMBASE (https://www.embase.com) to find articles published until the end of May 2024. No language restrictions were applied. One article was written in Chinese but the abstract was in English and we used a translation program for other necessary parts. Medical Subject Headings terms were used for searching PubMed and Cochrane, Emtree terms for EMBASE, and text keywords for identifying RCTs examining the effect of TXA on spine surgeries in patients who received blood transfusion (Supplementary Table S1).

### Study selection

Studies that (1) included patients who received blood transfusion during spine surgeries; (2) investigated interventions including TXA use; (3) provided comparisons between various methods of TXA administration and a control group (normal saline); and (4) measured outcomes as mean difference (MD) in intraoperative blood loss, postoperative blood loss, hemoglobin drop, blood transfusion rate, complication rate, and length of hospital stay in RCT studies were analyzed. Following the American Association of Hip Society, American Academy of Orthopaedic Surgeons, American Association of Hip and Knee Surgeons, and American Society of Regional Anesthesia and Pain Medicine guidelines (17), TXA treatments were divided into 10 groups: high-dose IV TXA (≥20 mg/kg or >1 g; IV_high), low-dose IV TXA (<20 mg/kg or ≤1 g; IV_low), low-dose IV TXA with temperature intervention (15 mg/kg; IV_low_temp), multiple IV TXA (multiple use of TXA pre- and post-surgery; IV_multi), high-dose topical TXA (>1.5 g; Topical_high), low-dose topical TXA (≤1.5 g; Topical_low), local infiltration (bilaterally administered into the paraspinal muscles before the incision; Local_inj), combined use (more than two administrations of TXA; Combine), oral (PO) TXA, and placebo.

Meanwhile, (1) reviews, abstracts, editorials, and letters that were not original articles; (2) non-RCTs; and (3) studies with noncomparable treatments were excluded. Three authors (SR Shim, S Han, and C Ihm) independently screened the titles and abstracts to identify relevant studies, reviewed the full text of articles for data extraction, and removed duplicates using Endnote software. All authors mutually discussed and resolved disagreements and cross-checked all references.

### Data extraction

Two authors (SR Shim and S Han) used a data extraction form to categorize the primary details of the studies (first author, publication year, region, and study design), patient characteristics (number of patients, age, type of surgery, and/or disease), and technical aspects (inclusion criteria and TXA treatments; Table 1).

**Table 1:** Characteristics of studies included in the systematic review.

Author	Region	Study design	Inclusion criteria	Treatment	Number of patients	Mean age (SD)
Fulin et al. 2023^a^	Asian	RCT (4-arm)	(1) Lumbar instability and failure of regular conservative treatment; (2) open fusion surgery	G1: Placebo, G2: Low dose intravenous use of TXA, G3: Low dose intravenous use of TXA with temperature intervention	232 (G1: 78, G2: 77, G3:77)	G1: 61.40 (3.34), G2: 61.87 (3.32), G3: 61.19 (3.19)
Dong et al. (24)	Asian	RCT (2-arm)	Laminectomy PLIF treatment	G1: Low dose intravenous use of TXA, G2: Multiple intravenous use of TXA	122 (G1: 63, G2: 59)	G1: 60.51 (9.30), G2: 58.66 (6.87)
Zhang et al. (25)	Asian	RCT (3-arm)	(1) Single-segment lumbar disc herniation, lumbar spine stenosis, and lumbar spondylolisthesis; (2) minimally invasive TLIF	G1: Low dose intravenous use of TXA, G2: High dose intravenous use of TXA, G3: Placebo	116 (G1: 39, G2: 39, G3: 38)	G1: 56.95 (11.41), G2: 55.67 (14.32), G3: 54.84 (10.62)
Yu et al. (26)	Western	RCT (2-arm)	Posterior thoracolumbar instrumented spine fusion	G1: Multiple intravenous use of TXA, G2: Oral use of TXA	261 (G1: 137, G2: 124)	G1: 64 (12), G2: 61 (13)
Hasan et al. (27)	Asian	RCT (2-arm)	Posterior spine fusion surgery	G1: High dose intravenous use of TXA, G2: Low dose intravenous use of TXA	166 (G1: 83, G2: 83)	G1: 14.1 (2.1), G2: 14.6 (3.0)
Shen et al. (28)	Asian	RCT (2-arm)	(1) Thoracolumbar burst fracture; (2) posterior internal fixation	G1: Low does topical use of TXA, G2: Placebo	76 (G1: 39, G2: 37)	G1: 38.85 (4.17), G2: 39.41 (6.51)
Dong et al. (29)	Asian	RCT (2-arm)	(1) AIS; (2) posterior spine fusion with segmental instrumentation	G1: Combination of intravenous and topical use of TXA, G2: High dose intravenous of TXA	80 (G1: 40, G2: 40)	G1: 14.4 (1.9), G2: 14.0 (2.0)
Zhang et al. (30)	Asian	RCT (3-arm)	(1) AIS; (2) PSS	G1: Combination of intravenous and oral use of TXA, G2: Multiple intravenous use of TXA, G3: High dose intravenous use of TXA	108 (G1: 36, G2: 36, G3: 36)	G1: 15.08 (1.71), G2: 15.14 (1.94), G3: 14.92 (2.02)
Arun-Kumar et al. (31)	Asian	RCT (4-arm)	Single or dual-level lumbar fixation with interbody fusions	G1: Placebo, G2: Local infiltration use of TXA, G3: Low dose intravenous use of TXA, G4: Low dose topical use of TXA	104 (G1: 26, G2: 26, G3: 26, G4: 26)	G1: 50.8 (3.4), G2: 48.0 (2.3), G3: 50.3 (3.2), G4: 51.9 (2.8)
Zhu et al. (32)	Asian	RCT (3-arm)	(1) Lumbar degenerative disease; (2) single-segment or double-segment posterior lumbar interbody fusion surgery	G1: Placebo, G2: Low dose intravenous use of TXA, G3: Multiple intravenous use of TXA	150 (G1: 50, G2: 50, G3: 50)	G1: 56.0 (9.5), G2: 54.8 (10.3), G3: 56.0 (9.9)
Li et al. (33)	Asian	RCT (4-arm)	(1) Two-level degenerative lumbar spine disease; (2) Two-level lumbar fusion	G1: Combination of topical and intravenous use of TXA, G2: Low dose intravenous use of TXA, G3: High dose topical use of TXA, G4: Placebo	280 (G1: 70, G2: 70, G3: 70, G4: 70)	G1: 65.28 (3.18), G2: 66.67 (3.27), G3: 65.61 (4.81), G4: 65.61 (3.17)
He et al. (34)	Asian	RCT (2-arm)	(1) Lumbar degenerative disease; (2) one- or two-level TLIF surgery	G1: Low dose intravenous use of TXA, G2: Placebo	40 (G1: 20, G2: 20)	G1: 57.95 (12.44), G2: 57.90 (11.76)
Xu et al. (35)	Asian	RCT (2-arm)	Spondylolisthesis, spondylolysis, severe spine instability, or large disc herniation	G1: Placebo, G2: Low dose topical use of TXA	60 (G1: 30, G2: 30)	G1: 50.6 (16.2), G2: 49.6 (12.8)
Wang et al. (36)	Asian	RCT (3-arm)	Thoracolumbar (T11–L2) single-vertebral A3–A4 subtype (Association of Internal Fixation Classification) fracture	G1: Low dose intravenous use of TXA, G2: High dose topical use of TXA, G3: Combination of intravenous and topical use of TXA	181 (G1: 61, G2: 61, G3: 59)	G1: 45.43 (8.18), G2: 45.72 (9.96), G3: 45.47 (11.24)
Yu et al. (37)	Western	RCT (2-arm)	Elective posterior thoracolumbar instrumented spine fusion	G1: Multiple intravenous use of TXA, G2: Oral use of TXA	83 (G1: 43, G2: 40)	G1: 64 (13), G2: 61 (12)
Ma et al. (38)	Asian	RCT (2-arm)	(1) Degenerative lumbar spine stenosis; (2) primary decompression and fusion surgery	G1: Low dose intravenous use of TXA, G2: Placebo	124 (G1: 62, G2: 62)	G1: 60.6 (4.7), G2: 61.2 (4.3)
Elmose et al. (39)	Western	RCT (2-arm)	Elective primary decompression or/and discectomy over 1 to 2 vertebral levels (without fusion or instrumentation)	G1: Low dose intravenous use of TXA, G2: Placebo	233 (G1: 117, G2: 116)	G1: 48.9 (15.4), G2: 51.1 (14.9)
Mu et al. (40)	Asian	RCT (3-arm)	Lumbar degenerative disease	G1: Low dose intravenous use of TXA, G2: Low dose topical use of TXA, G3: Placebo	126 (G1: 45, G2: 39, G3: 42)	G1: 54.20 (7.37), G2: 51.77 (8.13), G3: 52.57 (6.73)
Sudprasert et al. (41)	Asian	RCT (2-arm)	Posterior spine fusion with local autologous bone graft	G1: Low dose topical use of TXA, G2: Placebo	57 (G1: 29, G2: 28)	G1: 52.0 (5.5), G2: 51.5 (6.1)
Goobie et al. (42)	Western	RCT (2-arm)	(1) AIS; (2) elective posterior instrumented spine fusion	G1: Placebo, G2: High dose intravenous use of TXA	111 (G1: 55, G2: 56)	G1: 14.7 (1.8), G2: 14.9 (2.0)
Wang et al. (43)	Asian	RCT (2-arm)	Transforaminal thoracic interbody fusion	G1: Low dose intravenous use of TXA, G2: Placebo	80 (G1: 39, G2: 41)	G1: 41.2 (10.3), G2: 42.5 (9.5)
Nagabhushan et al. (44)^a^	Asian	RCT (4-arm)	Elective lumbar spine single level fusion surgery	G1: Low dose intravenous use of TXA, G2: Placebo	50 (G1: 25, G2: 25)	G1: 49.60 (9.79), G2: 51.72 (9.71)
Carabini et al. (45)	Western	RCT (2-arm)	Spine deformity correction	G1: Placebo, G2: Low dose intravenous use of TXA	61 (G1: 30, G2: 31)	G1: 68.0 (2.5), G2: 65.0 (1.7)
Kim et al. (46)^a^	Asian	RCT (3-arm)	(1) Lumbar spine stenosis; (2) PLIF	G1: Placebo, G2: Low dose intravenous use of TXA	48 (G1: 24, G2: 24)	G1: 65.2 (7.0), G2: 61.0 (9.0)
Seddighi et al. (47)	Asian	RCT (2-arm)	Major spine surgeries	G1: Low dose intravenous use of TXA, G2: Placebo	40 (G1: 20, G2: 20)	G1: 49.85 (12.21), G2: 43.70 (10.25)
Colomina et al. (48)	Western	RCT (2-arm)	Complex spine surgery	G1: Low dose intravenous use of TXA, G2: Placebo	95 (G1: 44, G2: 51)	G1: 59.20 (13.75), G2: 50.80 (14.25)
Basavaraj et al. (49)	Asian	RCT (2-arm)	Elective thoracic spine fixation surgery	G1: Low dose intravenous use of TXA, G2: Placebo	60 (G1: 30, G2: 30)	G1: 41.72 (15.578), G2: 38.50 (12.566)
Shi et al. (50)	Asian	RCT (2-arm)	(1) Lumbar spine stenosis or lumbar spondylolisthesis; (2) posterior lumbar decompression interbody fusion	G1: High dose intravenous use of TXA, G2: Placebo	96 (G1: 50, G2: 46)	G1: 53.76 (12.06), G2: 55.87 (13.14)
Liang et al. (51)^a^	Asian	RCT (3-arm)	Posterior lumbar decompression and fusion	G1: High dose topical use of TXA, G2: Placebo	60 (G1: 30, G2: 30)	G1: 60 (No data for SD), G2: 43 (No data for SD)
Peters et al. (52)^a^	Western	RCT (3-arm)	Posterior spine fusion	G1: Low dose intravenous use of TXA, G2: Placebo	32 (G1: 19, G2: 13)	G1: 51.13 (10.72), G2: 53.50 (10.26)
Verma et al. (53)^a^	Western	RCT (3-arm)	(1) AIS; (2) posterior spine arthrodesis	G1: Low dose intravenous use of TXA, G2: Placebo	83 (G1: 36, G2: 47)	G1: 15.30 (2.37), G2: 15.01 (2.37)
Wang et al. (54)	Asian	RCT (2-arm)	Degenerative lumbar instability with stenosis	G1: Placebo, G2: Low dose intravenous use of TXA	60 (G1: 30, G2: 30)	G1: 62.0 (4.6), G2: 63.1 (4.0)
Tsutsumimoto et al. (55)	Asian	RCT (2-arm)	Cervical laminoplasty	G1: Placebo, G2: Low dose intravenous use of TXA	40 (G1: 20, G2: 20)	G1: 65.8 (11.8), G2: 68.0 (11.0)
Farrokhi et al. 2011 (56)	Asian	RCT (2-arm)	Spine fixation surgery	G1: Placebo, G2: Low dose intravenous use of TXA	76 (G1: 38, G2: 38)	G1: 51.4 (11.6), G2: 45.5 (11.6)
Elwatidy et al. (57)	Asian	RCT (2-arm)	Spine operations	G1: High dose intravenous use of TXA, G2: Placebo	64 (G1: 32, G2: 32)	G1: 51.56 (19.08), G2: 49.75 (21.04)
Wong et al. (58)	Western	RCT (2-arm)	Elective posterior thoracic/lumbar instrumented spine fusions	G1: Low dose intravenous use of TXA, G2: Placebo	147 (G1: 73, G2: 74)	G1: 56.8 (16.2), G2: 50.0 (16.2)
Sethna et al. (59)	Western	RCT (2-arm)	Initial scoliosis correction	G1: High dose intravenous use of TXA, G2: Placebo	44 (G1: 23, G2: 21)	G1: 13.6 (1.8), G2: 14.0 (2.0)
Neilipovitz et al. (60)	Western	RCT (2-arm)	Posterior spine fusion	G1: Placebo, G2: Low dose intravenous use of TXA	40 (G1: 18, G2: 22)	G1: 13.7 (2.5), G2: 14.1 (2.1)

SD, standard deviation; RCT, randomized controlled trial; TXA, tranexamic acid; G1, group 1; G2, group 2; G3, group 3; G4, group 4; PLIF, posterior lumbar interbody fusion; TLIF, transforaminal lumbar interbody fusion; PSS, posterior spine fusion; AIS, adolescent idiopathic scoliosis.

^a^ Some groups of these studies had contained neither TXA nor Placebo so we excluded these groups.

We used the MD to analyze continuous outcomes, such as intraoperative blood loss, postoperative blood loss, hemoglobin drop, and length of hospital stay. To evaluate categorical outcomes, such as blood transfusion rate and complication rate, the odds ratio (OR) was determined. We calculated the pooled standard deviation using the pre- and post-standard deviations when these values were not provided for MD (18). As some articles described several median outcomes, Hozo's method was used to estimate the mean and standard deviation (19).

We calculated the hemoglobin drop as the difference between the preoperative levels and last measured postoperative values. DVT and PE were included in the analysis of complication rate.

### Quality assessment

The quality of studies was evaluated using the Cochrane Collaboration Risk of Bias 2.0 (RoB 2) tool (20). The following five domains were assessed: (1) randomization process, (2) deviations from intended interventions, (3) missing data, (4) outcome measurement, and (5) selection of the reported result.

Each domain was classified as “low,” “high” or “some concerns.” The overall risk of bias was classified as “low” when all domains were rated as “low” and “some concerns” when only one domain was rated as “some concerns.” If more than two domains were rated as “some concerns” or if any domain was rated as “high,” then the overall risk of bias was “high.”

### Network meta-analysis assessment of outcome findings and statistical analysis

For Bayesian NMA, specific graphical analysis was performed using the “gemtc” package in R software v.4.3.1 (R Foundation for Statistical Computing) (21). To compare the 10 TXA administration methods, a simulation was conducted by incorporating prior distributions and probabilities into the Markov Chain Monte Carlo (MCMC). Subsequently, the optimal convergence model was selected by reviewing the trace plot, normal distribution plot, and the MCMC standard error of the generated posterior distribution. Thus, the posterior probability of the effect size for each treatment was calculated. A consistency test between the direct and indirect comparisons was performed using node-splitting assessments. We used funnel plot and Egger test to assess publication bias (22).

In the Bayesian approach, the optimal probability of selecting individual treatments is determined using the generated posterior distribution. This distribution reflects the priority of each treatment, represented as the surface under the cumulative ranking curve (SUCRA); a higher SUCRA value indicates a higher rank of the intervention (21, 23). The analysis pooled the MDs and 95% credible intervals (CrI). A two-sided *p*-value of ≤.05 or a 95% CrI that does not contain a null value (MD = 0) was considered significant.

## Results

### Study selection

**Figure 1:**
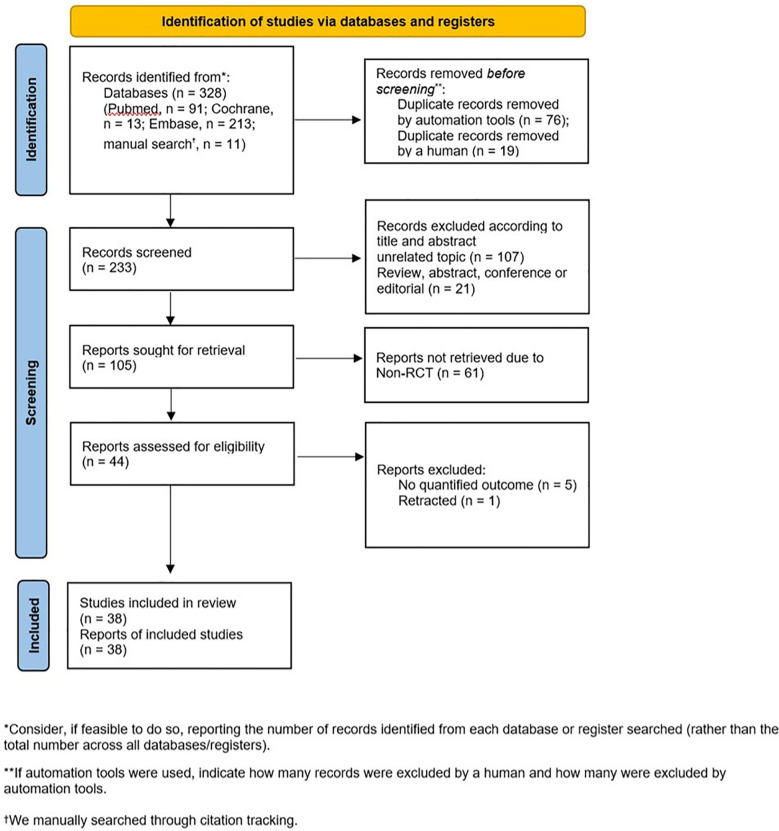
PRISMA flowchart detailing the study selection process.

The primary search identified 317 articles from various electronic databases, including PubMed (*n* = 91), Cochrane (*n* = 13), and EMBASE (*n* = 213), with an additional 11 articles identified during manual search through citation tracking. We excluded 95 articles with duplicate or overlapping data, 107 articles with unrelated topics, 21 articles that were not original research, and 61 articles that were not RCTs. After a full-text review of 44 articles, six articles were further excluded: one had been retracted, while the rest had no quantified outcome. After the final selection, 38 articles were included for data extraction (Figure 1).

In total, 3,886 patients were included in 38 studies (12, 24–60), with approximately 55% being women. All studies were RCTs conducted in various regions: 27 studies were performed in Asia, while 11 studies were conducted in Western countries (Table 1). Of the included studies, 25 employed a two-arm design, while nine studies employed a three-arm design. However, four of the nine studies were classified as two-arm trials as some groups did not use TXA or a placebo. Four studies were designed as four-arm trials. However, two of these studies did not use TXA or a placebo. Hence, one was classified as a two-arm study, while the rest were classified as 3-arm studies.

### Inconsistency test

The inconsistency tests for the NMA assumption were conducted using the node-splitting approach. The findings (*p* > 0.05) indicated consistency across direct and indirect comparisons for all outcomes.

### Quality assessment

**Figure 2:**
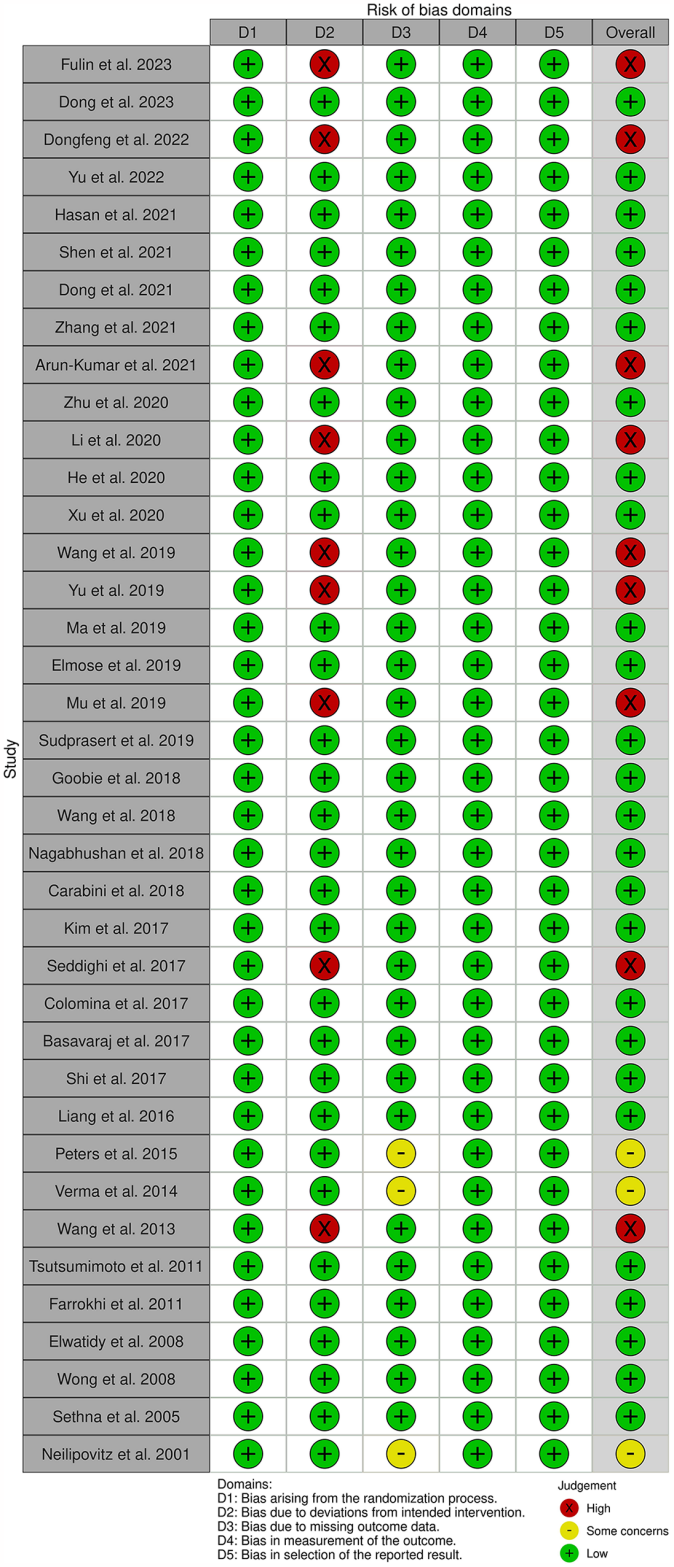
Risk of bias assessment.

All studies were evaluated using the RoB 2 tool. The randomization process (D1) was recorded as “low” (Figure 2). However, nine studies were classified as having a “high” risk for deviations from intended interventions (D2) due to the lack of blinding. For missing data (D3), three studies were rated as “some concerns” as the number of patients between the TXA group and placebo group differed by >10%. By contrast, the outcome measurement (D4) and selection of the reported result (D5) were both rated as “low.”

### Outcome findings

**Figure 3:**
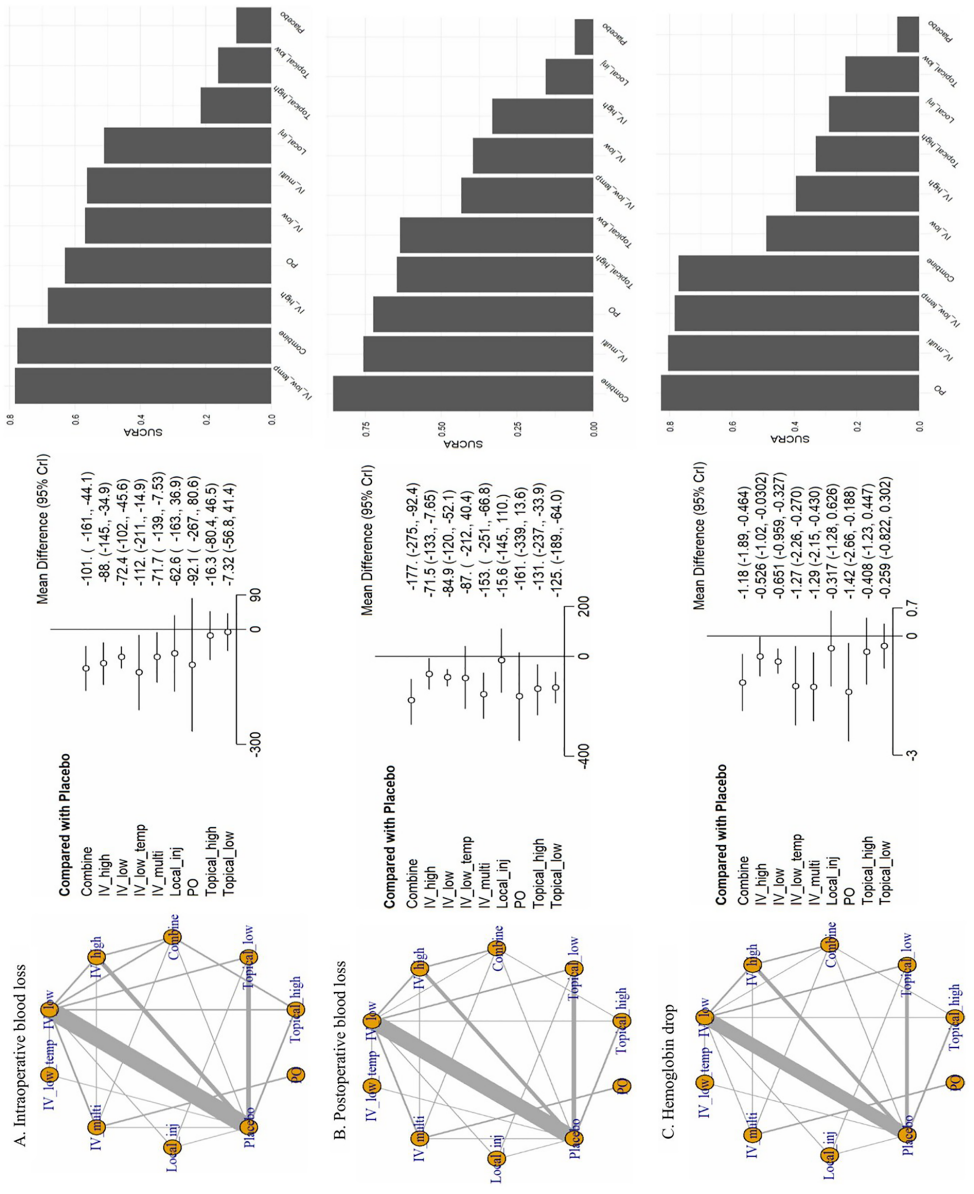
Network plots from the network meta-analysis. **(A)** Intraoperative blood loss, **(B)** postoperative blood loss, **(C)** Hemoglobin drop. CrI, credible intervals; Combine, more than two administrations of TXA; IV_high, high-dose IV TXA (≥20 mg/kg or >1 g); IV_low, low-dose IV TXA (<20 mg/kg or ≤1 g); IV_low_temp, low-dose IV TXA with temperature intervention (15 mg/kg); IV_multi, multiple use of TXA pre- and post-surgery; Local_inj, local infiltration (bilaterally administered into the paraspinal muscles before the incision; PO, oral; Topical_high, high-dose topical TXA (>1.5 g); Topical low, low-dose topical TXA (≤1.5 g).

**Figure 4:**
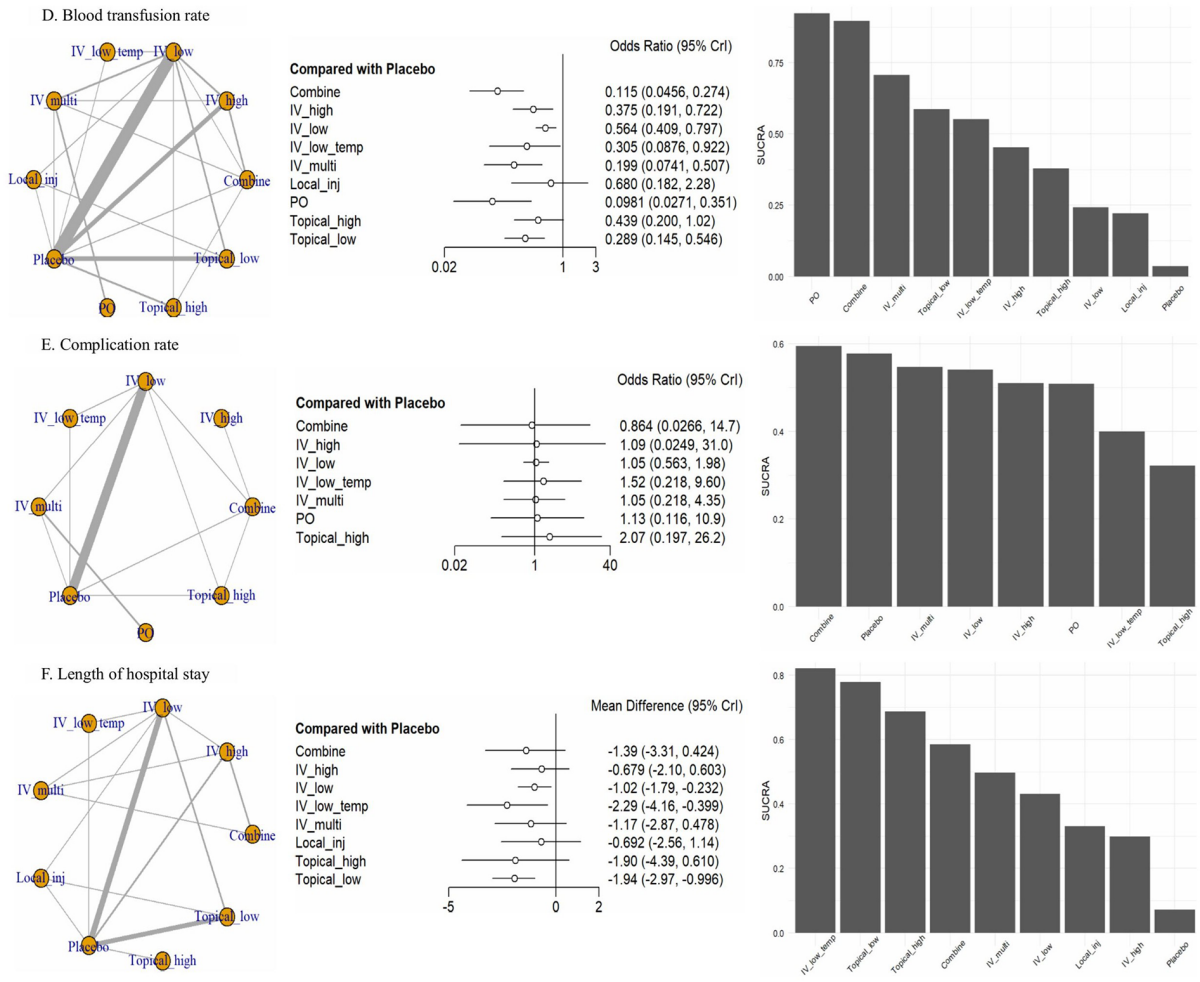
Network forest plot. **(D)** Blood transfusion rate, **(E)** complication rate, **(F)** length of hospital stay. CrI, credible intervals; Combine, more than two administrations of TXA; IV_high, high-dose IV TXA (≥20 mg/kg or >1 g); IV_low, low-dose IV TXA (<20 mg/kg or ≤1 g); IV_low_temp, low-dose IV TXA with temperature intervention (15 mg/kg); IV_multi, multiple use of TXA pre- and post-surgery; Local_inj, local infiltration (bilaterally administered into the paraspinal muscles before the incision; PO, oral; Topical_high, high-dose topical TXA (>1.5 g); Topical low, low-dose topical TXA (≤1.5 g).

Through this analysis, we organized six outcomes (Figures 3, 4): intraoperative blood loss, postoperative blood loss, hemoglobin drop (Figure 3), blood transfusion rate, complication rate, and length of hospital stay (Figure 4).

For intraoperative blood loss, we included 36 studies involving 10 treatments. IV_low_temp (MD: −112.0, 95% CrI: −211.0 to −14.9), Combine (MD: −101.0, 95% CrI: −161.0 to −44.1), and IV_high (MD: −88.0, 95% CrI: −145.0 to −34.9) were significantly different compared with placebo. The following SUCRA rankings were consistent with the abovementioned findings: IV_low_temp (SUCRA: 78.37%) was the highest ranked, followed by Combine (SUCRA: 77.65%) and IV_high (SUCRA: 68.32%). On the contrary, Topical_high (SUCRA: 21.51%) and Topical_low (SUCRA: 16.17%) were the lowest ranked.

For postoperative blood loss, we analyzed 32 studies involving 10 treatments. Compared with placebo, Combine (MD: −177.0, 95% CrI: −275.0 to −92.4) and IV_multi (MD: −153.0, 95% CrI: −251.0 to −66.8) showed significant differences. SUCRA rankings were consistent, with Combine (SUCRA: 85.66%) and IV_multi (SUCRA: 75.67%) treatments achieving the highest rank, whereas Local_inj (SUCRA: 15.67%) achieved the lowest rank.

Regarding the hemoglobin drop, we analyzed 28 studies involving 10 treatments. PO (MD: −1.42, 95% CrI: −2.66 to −0.19), IV_multi (MD: −1.29, 95% CrI: −2.15 to −0.43), IV_low_temp (MD: −1.27, 95% CrI: −2.26 to −0.27), and Combine (MD: −1.18, 95% CrI: −1.89 to −0.46) showed significant differences compared with placebo. SUCRA rankings corroborated these results with the most effective being ranked in the following order: PO (SUCRA: 82.81%), IV_multi (SUCRA: 80.53%), IV_low_temp (SUCRA: 78.43%), and Combine (SUCRA: 77.13%).

For blood transfusion rate, we included 28 studies involving 10 treatments. PO (OR: 0.10, 95% CrI: 0.03–0.35), Combine (OR: 0.12, 95% CrI: 0.05–0.27), IV_multi (OR: 0.20, 95% CrI: 0.07–0.51), Topical_low (OR: 0.29, 95% CrI: 0.15–0.55), and IV_low_temp (OR: 0.31, 95% CrI: 0.09–0.92) were significantly superior to placebo. SUCRA rankings confirmed these findings, with PO (SUCRA: 92.38%), Combine (SUCRA: 89.68%), and IV_multi (SUCRA: 70.77%) ranking the highest, followed by Topical_low (SUCRA: 58.75%) and IV_low_temp (SUCRA: 55.23%), whereas Local_inj (SUCRA: 22.16%) was ranked the lowest.

In terms of complication rate, we analyzed 14 studies involving eight treatments, excluding Topical_low and Local_inj. However, the differences between the treatments were not significant compared with placebo.

In terms of the length of hospital stay, we included 19 studies involving nine treatments, excluding PO. Significant differences were only observed for IV_low_temp (MD: −2.29, 95% CrI: −4.16 to −0.40) and Topical_low (MD: −1.94, 95% CrI: −2.97 to −0.10) compared with placebo. SUCRA rankings indicated that IV_low_temp (SUCRA, 82.12%) was the highest ranked, followed by Topical_low (SUCRA: 77.86%).

Overall, the SUCRA values for ranking probabilities across outcomes indicated that TXA achieved the highest overall ranking in the NMA (Figures 3, 4).

### Publication bias

Statistical methods for detecting publication bias or small-study effects are shown in Supplementary Figure S1. Funnel plots for individual outcomes (intraoperative blood loss, postoperative blood loss, hemoglobin drop, blood transfusion rate, complication rate, and length of hospital stay) showed visual symmetry. Additionally, Egger's regression analysis did not reveal any evidence of publication bias or small-study effects in this NMA (*p* > 0.05).

## Discussion

This systematic review and NMA is the first study to evaluate the effects of low-dose IV TXA combined with temperature intervention on blood transfusion outcomes. We aimed to evaluate the effects of various TXA administration methods on blood loss, blood transfusion rate, complication rate, and length of hospital stay during spine surgery. A comprehensive NMA that closely examined 38 RCTs showed that IV_low_temp and Combine significantly improved surgical indices such as blood loss, hemoglobin drop, and blood transfusion rate. TXA has been used in various surgical procedures, demonstrating potential postoperative hemostatic benefits along with reduced blood loss and transfusion rates (61, 62). Additionally, TXA may aid in bleeding control in patients with platelet function abnormalities (63). To minimize the risk of side effects and maximize the benefits of TXA, the optimal dose and route must be carefully considered.

Various methods for administering TXA exist, with IV administration being the most commonly used in our study. IV TXA demonstrated a significant reduction in intraoperative blood loss (MD: −185.0 ml, 95% CrI: −302.1 to −67.9) (10) and estimated blood loss compared with placebo (841 vs. 1,336 ml, *p* = .002) (15). In our study, IV TXA also showed a reduction in most outcomes (intraoperative blood loss, postoperative blood loss, hemoglobin drop, and blood transfusion rate), positioning it as a top-priority method for TXA administration during blood transfusion. The time needed to achieve the maximum plasma levels of TXA is 30 min post-intramuscular administration, 2 h post-oral administration, and 5–15 min post-IV administration (64); therefore, IV is the recommended method.

With regard to IV doses, low-dose IV TXA has been the most commonly used (61), likely due to concerns about complications such as MI, VTE, and PE. Low-dose IV TXA reduced blood loss by 34% (*p* < 0.001). Meanwhile, evidence related to the effect of high-dose TXA remains lacking (11). Additionally, the low-dose IV TXA group experienced significantly reduced blood loss and required significantly fewer blood transfusions compared with the control group, with no significant differences in intraoperative and postoperative complications in patients with adolescent idiopathic scoliosis undergoing posterior spine fusion (65). In our study, low-dose IV TXA showed significant effects on five outcomes (intraoperative blood loss, postoperative blood loss, hemoglobin drop, blood transfusion rate, and length of hospital stay). It reduced intraoperative blood loss (940 ml vs. 1,280 ml, *p* = 0.01) (48), postoperative blood loss (29.9%, *p* < 0.01) (54), and the total amount of blood transfused (28%) compared with placebo (*p* = 0.045) (60). Additionally, high-dose IV TXA showed significant effects on four parameters (intraoperative blood loss, postoperative blood loss, hemoglobin drop, and blood transfusion rate). This treatment resulted in less intraoperative blood loss (836 ± 373 vs. 1,031 ± 484 ml, *p* = 0.02) (42), a 49% reduction in intraoperative blood loss (*p* < 0.007) and an 80% decrease in blood transfusion requirements (*p* < 0.008) compared with placebo (57). However, high-dose TXA showed no additional benefits (11). Consequently, low-dose TXA has more pronounced benefits with a lower risk of complications.

In addition to the TXA injection route or dose, other factors should also be considered to improve surgical outcomes. The results of our study demonstrated that the combination of low-dose IV TXA and temperature intervention (IV_low_temp) is effective in reducing intraoperative blood loss. Compared with a placebo, it showed an MD of −112.0 ml (95% CrI: −211.0 to −14.9) and ranked first in SUCRA (78.37%). This finding further underscores the significance of maintaining the core body temperature of patients. In particular, Li et al. (12) randomly assigned patients undergoing spine fusion, with one group receiving low-dose IV TXA combined with temperature control to maintain the body temperature above 36°C. Axillary temperature was monitored to assess the changes in body temperature. Warming measures included controllable electric heating blankets, fluid warmers, and operating room temperature control (24°C). This group showed superior efficacy in reducing intraoperative blood loss by −102.48 ± 141.876 ml (95% CrI: −147.152 to −57.808) (*p* < 0.001), decreasing hemoglobin levels by −1.25 ± 0.727 g/dl (95% CrI: −1.479 to −1.021), and length of hospital stay by −2.37 ± 0.333 d (95% CrI: −3.024 to −1.716). These findings align with our results, underscoring that temperature control enhances the hemostatic properties of TXA, effectively reducing intraoperative bleeding and subsequently improving patient outcomes. Hypothermia primarily impairs platelet function by disrupting the release of thromboxane A2, which is essential for initial platelet plug formation (66). Anesthetics inhibit the body's thermoregulation, and the low temperature in the operating rooms increases the risk of hypothermia (67). Even mild hypothermia (<1°C) significantly increased blood loss by approximately 16% and the relative risk of blood transfusion by approximately 22% (68). Maintaining perioperative normothermia effectively reduces these risks by clinically significant amounts. Therefore, this treatment has significant potential to minimize blood loss, making it one of the most effective strategies for reducing surgical risks. Further studies are needed to explain the effects of TXA on temperature management during surgery and establish standardized protocols for its use in spine surgery.

Furthermore, we defined Combine as more than two administrations of TXA that effectively and significantly reduced both intraoperative and postoperative blood loss. Compared with placebo, the MDs were −101.0 ml (95% CrI: −161.0 to −44.1) for intraoperative blood loss and −177.0 ml (95% CrI: −275.0 to −92.4) for postoperative blood loss. The significance of combined TXA administration in reducing blood loss and transfusion rates aligns with the findings of a previous study (13). The previous study only included IV and topical administration, whereas our study includes IV, topical, and PO administration, providing flexibility through various combination administration routes. Combining IV and topical TXA yields superior outcomes in reducing total blood loss and allogeneic transfusion rates (69). The combined use of TXA can stabilize the fibrinolytic system in the first 24 h after surgery, thus efficiently reducing blood loss (33). This approach indicates that maximizing the blood-clotting effect can be achieved using various TXA administration routes or doses.

Our study has some limitations. First, several individual RCTs were of low quality due to the absence of blinding. However, the overall quality assessment was deemed good as all studies employed randomization. Second, this NMA was unable to establish a closed loop owing to insufficient data, resulting in a synthesis encompassing all types of spine surgeries rather than conducting a detailed subgroup analysis of specific categories, such as cervical, lumbar, thoracic, sacral, and coccygeal surgeries. Third, this comprehensive systematic review and NMA, which integrates findings from various studies, provides an integrated perspective on TXA. However, the high heterogeneity in the study designs and patient demographics potentially influences the overall conclusions; consequently, careful consideration of methodological concerns is essential.

Evaluation of safety issues related to TXA administration, such as the risks of stroke, MI, DVT, and PE, showed that TXA did not significantly increase the incidence of these complications. Therefore, TXA can be safely used in spine surgery and effectively reduces perioperative bleeding. Our study also found that all TXA administration routes were well tolerated and safe compared with placebo.

## Conclusion

Low-dose intravenous TXA with temperature intervention and the combined use of TXA significantly improved blood loss, hemoglobin drop, and blood transfusion rate during spine surgery. Further research involving a larger population and prospective design is needed to accurately quantify the effect of TXA on blood transfusion rates in the current surgical practices. Additionally, careful evaluation of TXA as a potential risk factor for complications is essential.

## Data Availability

The original contributions presented in the study are included in the article/Supplementary Material, further inquiries can be directed to the corresponding author.
