# Supplementary material for: Effect of tranexamic acid in spine surgeries: a systematic review and network meta-analysis
Source: Front Surg. 2025 Apr 11;12:1550854. doi: 10.3389/fsurg.2025.1550854 (PMC12021860; doi:10.3389/fsurg.2025.1550854)

**Supplementary appendix**

This supplemental material has been provided by the authors to give readers additional information about their work.

Supplementary Table S1. Search queries

Supplementary Figure S1. Funnel plots for publication bias

**Supplementary Table S1. Search queries**

| **PubMed and Cochrane Library** | |
| --- | --- |
|  | 1. Population  ("Blood Transfusion"[Mesh] OR "Blood Transfusion*"[tiab]) AND ("Spinal Fusion"[Mesh] OR "Spinal Fusion"[tiab] OR "Spinal surgery"[tiab]) |
|  | 2. Intervention  ("Tranexamic Acid"[Mesh]OR "Tranexamic Acid"[tiab])  3. Species  ("humans"[MeSH Terms]) |
|  |  |
| **Embase** | |
|  | 1. Population  ('blood transfusion'/exp OR 'blood transfusion':ti,ab) AND ('spine fusion'/exp OR 'spinal fusion':ti,ab OR 'spinal surgery':ti,ab) |
|  | 2. Intervention  ('tranexamic acid'/exp OR 'tranexamic acid':ti,ab)  3. Species  ('human'/de) |

**Supplementary Figure S1. Funnel plots for publication bias**

a. Intraoperative blood loss b. Postoperative blood loss


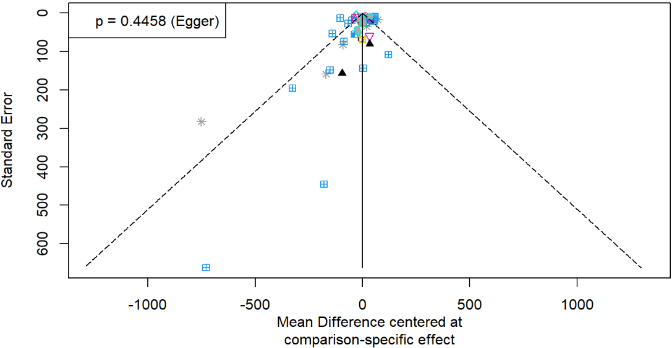

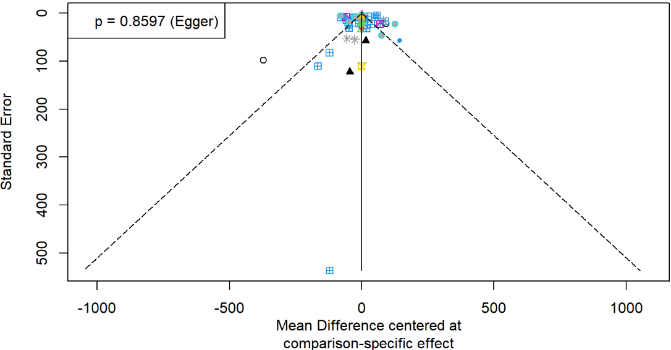


c. Hemoglobin drop d. Blood transfusion rate


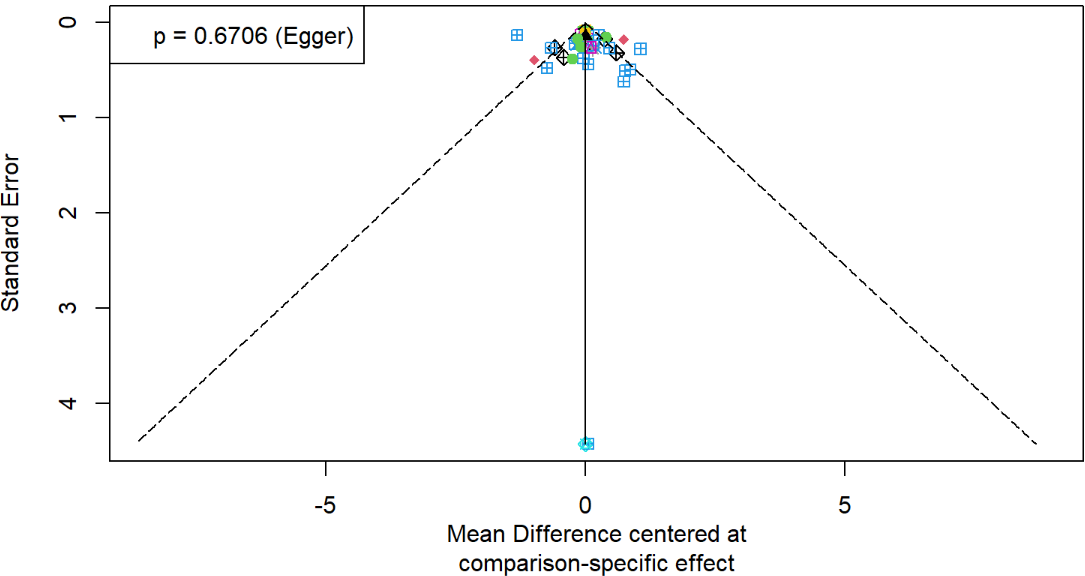

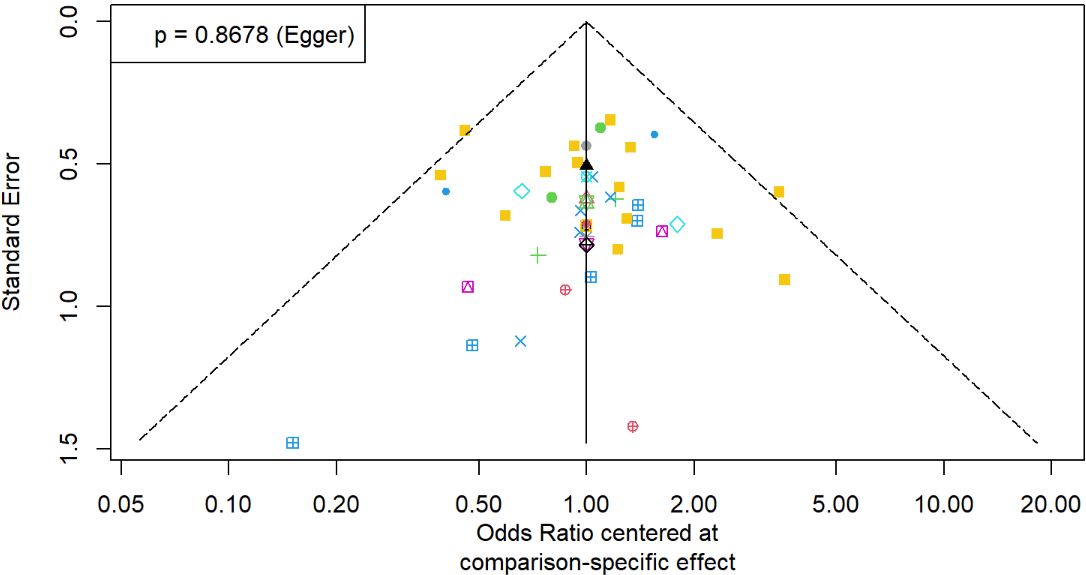


e. Complication rate f. Length of hospital stay


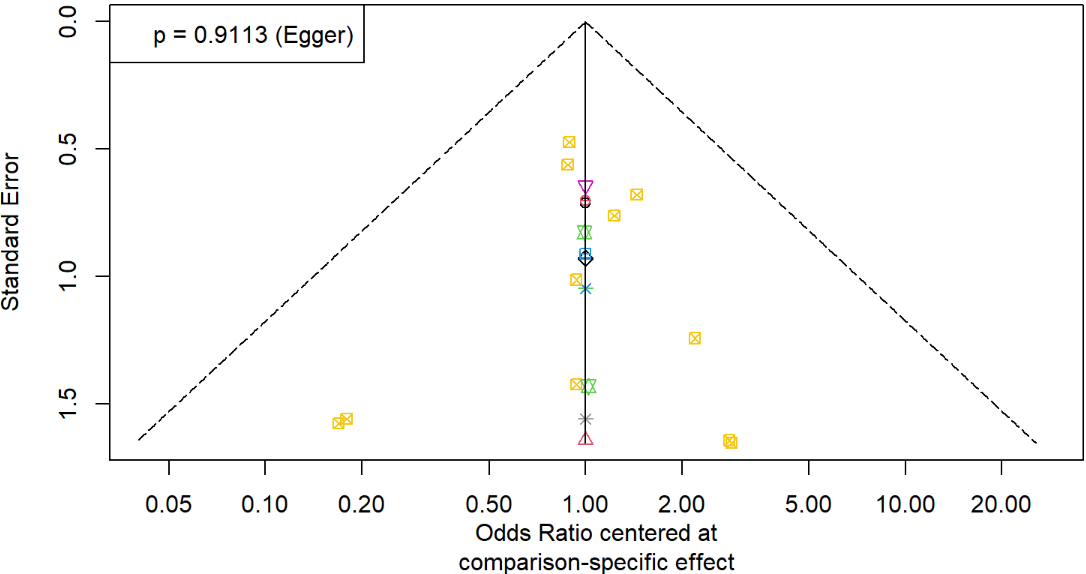

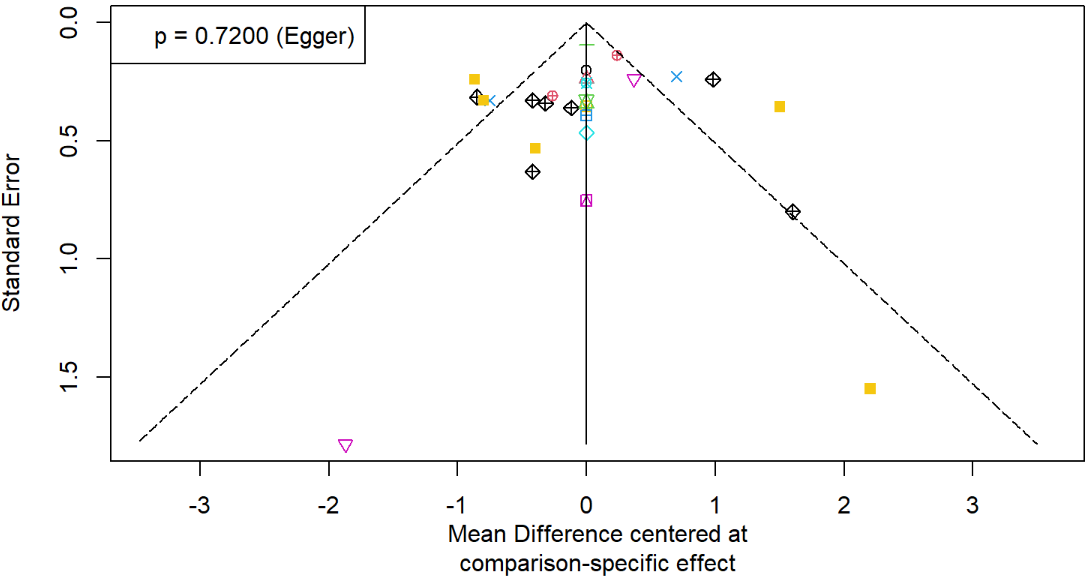

Supplement: Supplementary file 1 [file Table1.docx]
